# Bank Resolution Trade-Offs Under Coupled Liquidity and Credit Risks: An Agent-Based Network Analysis of Systemic Stability

**DOI:** 10.3390/e28060618

**Published:** 2026-05-31

**Authors:** Qianqian Gao, Hongjie Pan, Yinglin Liu, Naixi Chen

**Affiliations:** 1 School of Financial Technology, Shanghai Lixin University of Accounting and Finance, Shanghai 201209, China; gaoqianqian@lixin.edu.cn (Q.G.); 20220003@lixin.edu.cn (Y.L.); 2 Business School, Ningbo University, Ningbo 315211, China; 3 School of Economics and Management, Liaoning Petrochemical University, Fushun 113001, China; chennaixi@lnpu.edu.cn

**Keywords:** complex financial networks, systemic stability, agent-based modeling, liquidity-credit shocks

## Abstract

Prolonged downturns in the global economy have simultaneously increased banks’ credit risk exposures and intensified the need for effective liquidity management. This study develops a dynamic agent-based financial network comprising banks, depositors, firms, and the central bank to examine trade-offs in bank resolution under coupled liquidity and credit risks from the perspective of systemic stability. The simulation results show that, for liquidity risk management, when banks adopt the asset-sale strategy, both default probability and expected returns in the banking system exhibit a nonlinear pattern: they first decline and then rise as the asset depreciation ratio increases. Furthermore, at moderate levels of asset depreciation, the asset-sale strategy helps preserve heterogeneity within the banking system, thereby preventing excessive risk concentration, and performs better than the liability-expansion strategy. Regarding credit risk resolution, the debt-relief strategy significantly improves systemic stability, whereas the effectiveness of the debt-extension strategy depends critically on liquidity management conditions. Under liability-expansion scenarios, default risk initially declines but later rises as debt maturity is extended, whereas expected returns move in the opposite direction. Under asset-sale conditions, the debt-extension strategy enhances systemic stability only when the allowable number of debt extensions is sufficiently high. The analysis of strategic trade-offs indicates that combining the debt-relief strategy with the asset-sale strategy generates a positive synergistic effect and strengthens systemic resilience, whereas the interaction between the debt-extension and asset-sale strategies produces offsetting effects. These findings offer useful implications for banks and regulators in designing coordinated and adaptive frameworks for risk resolution and systemic stability.

## 1. Introduction

Banks’ liquid assets are central to their operations and overall value. Liquidity shortfalls can quickly escalate into systemic threats [1]. Since the 2008 global financial crisis, liquidity risk has attracted increasing regulatory attention [2].

At the same time, banks continue to face persistent frictions in credit markets, particularly moral hazard [3], as borrower defaults can impose substantial losses on banks and undermine systemic stability. Credit risk, a core component of financial risk, affects both banking performance and the broader economy [4,5].

Meanwhile, greater global interconnectedness has amplified the contagion potential of financial risks [6,7]. Economic headwinds have further heightened credit risk and intensified pressures on liquidity management. As a cornerstone of financial stability, the banking sector’s response to simultaneous liquidity and credit challenges is therefore critical [8,9,10]. Against this backdrop, this study models the behavior and interactions of banks, depositors, firms, and the central bank within a dynamic, multi-agent financial network. It simulates liquidity shocks and credit defaults, evaluates how alternative resolution strategies affect the stability of the banking system under both types of risk, and examines the interactions between these strategies.

A substantial body of research has examined liquidity risk during periods of financial stress. Cornett et al. [1], Adrian and Shin [11], and Ratnovski and Huang [12] examine balance-sheet dynamics under stress. Acharya and Mora [13] and Choudhary and Limodio [14] show that elevated liquidity risk can constrain credit supply, while Iyer et al. [15] find that central bank support may induce liquidity hoarding. From the liquidity risk management perspective, Grundke and Kühn [16] and Ferrara et al. [17] show that liquidity requirements and macroprudential policies can reduce default risk and mitigate vulnerabilities in the financial system to liquidity shocks. Thakor [18] further underscores the importance of bank capital and solvency risk in preserving financial stability. Liu et al. [10] show that the liquidity coverage ratio plays a central role in shaping the stability of China’s interbank network, suggesting that adjustments to this ratio can help contain liquidity risk. A parallel strand of research focuses on credit risk and the mechanisms that shape bank lending and risk-taking. Loose monetary conditions tend to encourage risk-taking [19,20], whereas diversified lending relationships may mitigate credit risk [21,22]. Other studies point to adverse selection in bank–firm credit relationships [23,24] and systemic factors that influence non-performing loans [25]. A related strand of research examines how credit risk management instruments affect bank behavior. Arping [26] and Buston [27] highlight the potentially ambiguous effects of credit default swaps (CDSs). Arping [26] argues that CDS can discipline borrowers by making lenders’ threats to terminate loans more credible, but may also weaken borrowers’ incentives to exert effort by strengthening lenders’ bargaining position. Buston [27], in contrast, shows that credit protection trading can increase banks’ incentives to take risk, while also improving the capacity of credit supply to absorb macroeconomic fluctuations. Extending the discussion to broader credit risk transfer (CRT) instruments, Beyhaghi et al. [28] find that banks facing tighter capital and liquidity constraints are more likely to use such instruments to manage their loan portfolios. Gersbach and Rochet [29] further provide a theoretical basis for countercyclical capital requirements.

Beyond the strand of research that examines liquidity risk and credit risk in isolation, a growing body of literature has turned to examining the interrelationship between the two risks and its implications for bank stability. Some studies evaluate their relative importance during periods of financial crisis by analyzing the determinants of interbank market spreads. Gefang et al. [30], De Socio [31], and Beirne [32] show that both liquidity risk and credit risk affect market spreads, although their relative contributions differ across crisis phases, market environments, and maturities. Gefang et al. [30] find that short-term spreads are driven primarily by liquidity risk, whereas long-term spreads tend to reflect credit-risk considerations to a greater extent. De Socio [31] shows that credit risk had already risen before the crisis, while liquidity risk became the dominant source of widening Euribor spreads after the crisis. Beirne [32] likewise reports that both forms of risk significantly affected EONIA spreads during the crisis. Ippolito et al. [33] further demonstrate that, following the freeze in the European interbank market, banks were exposed to simultaneous runs on both sides of their balance sheets, suggesting that liquidity and credit risks can become mutually reinforcing under adverse shocks and thereby increase banks’ fragility.

The literature, however, has not reached a consensus on whether the relationship between liquidity risk and credit risk is stable over time. Imbierowicz and Rauch [34] find no economically significant synchronization or lagged relationship between the two risks. They nevertheless show that each risk independently raises the likelihood of bank default, while their interaction depends on banks’ overall risk profiles. By contrast, Chen and Lin [35] and Bouslimi et al. [36] show a significant association between liquidity and credit risks, with Bouslimi et al. [36] further identifying a nonlinear threshold effect. From the perspective of systemic stability and operational outcomes, Kesraoui et al. [9], Aydemir and Guloglu [37], Djebali and Zaghdoudi [38], and Abdesslem et al. [39] provide consistent evidence that these two risks jointly shape bank stability, default probability, and interest-rate spreads, and that their effects exhibit nonlinear or phase-dependent patterns.

Overall, existing studies suggest that liquidity risk and credit risk may jointly affect the resilience of the banking system. However, the literature remains divided on whether their interaction follows a stable and generalizable mechanism. In particular, the absence of a significant synchronous interaction documented by Imbierowicz and Rauch [34] should not be interpreted as evidence that the two risks cannot become coupled. Rather, such coupling may arise only under specific shock configurations, behavioral responses, or network contagion channels. This suggests that the relationship between liquidity and credit risks is state-dependent rather than static and may therefore not be fully captured by empirical approaches based on static specifications or average effects. Such approaches may overlook the endogenous dynamics generated by risk management decisions, behavioral feedback among agents, and the structure of interbank networks.

Departing from the empirically oriented studies discussed above, another strand of the literature employs complex network and agent-based modeling (ABM) frameworks to investigate how financial risks propagate through inter-agent linkages and multiple transmission channels. Zhang et al. [40], Chen et al. [41], Denbee et al. [42], and Chen [43] use network models to analyze risk in interbank networks, showing that interbank interdependence and its dynamic evolution can intensify risk propagation. These studies provide important insights into the mechanisms through which shocks are transmitted across interbank markets. Bookstaber et al. [44] extend this perspective by developing a multi-agent financial network model, demonstrating that both network structure and behavioral heterogeneity are central to the emergence of systemic risk. A related body of research extends the analysis to multilayer networks and multiple contagion channels. Paltalidis et al. [45], Silva et al. [46], Li et al. [47], Hu et al. [48], and Gao and Fan [49] move beyond single-layer interbank exposures by incorporating additional layers such as bank-firm credit relations, asset-price dynamics, sovereign credit risk, and heterogeneous agent behavior. Their findings indicate that sovereign credit risk may spread rapidly and that network topology, cross-layer exposures, and indirect linkages can substantially reinforce contagion. They also show that policy effectiveness may vary markedly across multilayer networks. Furthermore, Tedeschi et al. [50], Gurgone and Iori [51], and Adão et al. [52] examine how debt restructuring, macroprudential regulation, and monetary policy affect financial stability within agent-based frameworks. Their results suggest that banks’ tolerance for firm debt distress, the performance of prudential regulation under network heterogeneity, and the impact of interest-rate changes on banks’ risk-taking and liquidity management all operate through adjustments in agents’ micro-level behavior and affect macro-financial stability.

Despite these contributions, the literature has several limitations. Empirical studies often rely on historical data and regression methods, which may not fully capture the dynamic complexity of financial networks or the endogenous behavior of agents within them. Although network models and agent-based models can provide richer structural insights, existing research on single-layer financial networks typically focuses on one type of financial linkage, such as interbank or bank–firm relationships, to examine the characteristics of a particular financial risk and its effects on the financial network as a whole. This reliance on a single source of risk makes it difficult to capture simultaneously liability-side pressure from deposit runs, asset-side credit losses from firm defaults, and the feedback loops that arise between the two through banks’ behavioral adjustments. Furthermore, existing studies that consider multiple risks have primarily focused on the transmission mechanisms of multi-risk channels, without explicitly modeling banks’ strategic responses to dual-risk shocks. Consequently, it is difficult to examine the nonlinear effects of banks’ risk resolution strategies on systemic stability under the joint pressures of liquidity and credit risks.

The studies most closely related to this paper are Tedeschi et al. [50] and Adão et al. [52], both of which employ multi-agent modeling frameworks to examine how bank behavior affects financial stability. Adão et al. [52] examine how changes in interest rates affect banks’ risk-taking in loan portfolios, whereas Tedeschi et al. [50] analyze banks’ debt restructuring responses to financial delinquency among firms and the resulting macroeconomic consequences. These studies provide valuable insights into the behavioral foundations of financial stability. However, their modeling frameworks are largely centered on a single dominant source of risk and a relatively specific transmission context. As a result, they do not explicitly account for situations in which banks are simultaneously exposed to credit losses on the asset side and liquidity pressures on the liability side under compound risk conditions. Nor do they examine how asset-side and liability-side resolution strategies may interact, conflict, and jointly shape systemic outcomes.

To address these limitations, this paper develops a multi-agent financial network that extends single-layer representations by incorporating heterogeneous agents and multiple interaction linkages, while treating liquidity and credit risks as coupled pressures on the asset and liability sides of banks’ balance sheets. Specifically, credit risk arises from firm defaults and affects the asset side of banks’ balance sheets, whereas liquidity risk stems from depositor withdrawals and affects the liability side. This structure allows us to introduce a set of bank resolution strategies tailored to different risk scenarios. Under liquidity pressure, banks may respond by shrinking assets or expanding liabilities; under credit risk shocks, they may grant debt extensions or provide debt relief. The framework thus makes it possible to assess not only the separate effects of individual resolution strategies, but also the strategic interactions and trade-offs that emerge when these measures are deployed under joint liquidity and credit stress.

This paper makes three main contributions. First, it integrates liquidity shortfalls and credit defaults within a unified multi-agent financial network framework. By explicitly modeling the interactions among heterogeneous agents in real-world financial systems, the framework provides a more transparent representation of these interactions. It simultaneously captures pressures on the asset and liability sides of banks’ balance sheets, together with the associated risk transmission processes, thereby making it possible to identify the systemic consequences of banks’ risk resolution strategies. Most importantly, the framework provides a basis for examining the interactions among different risk resolution strategies that are difficult to detect in single-layer network models or single-channel models. Second, the paper identifies the endogenous trade-offs between banks’ asset-side and liability-side risk resolution strategies under coupled liquidity and credit risks and shows that different strategies, as well as their combinations, may have nonlinear effects on systemic stability. Third, in addition to default probability and expected return on capital, this paper introduces a Shannon entropy-based measure of bank states, which classifies banks into three states—normal, liquidity distress, and default—thereby making it possible to characterize the dispersion and uncertainty of the banking system’s state distribution.

## 2. The Model

**Figure 1:**
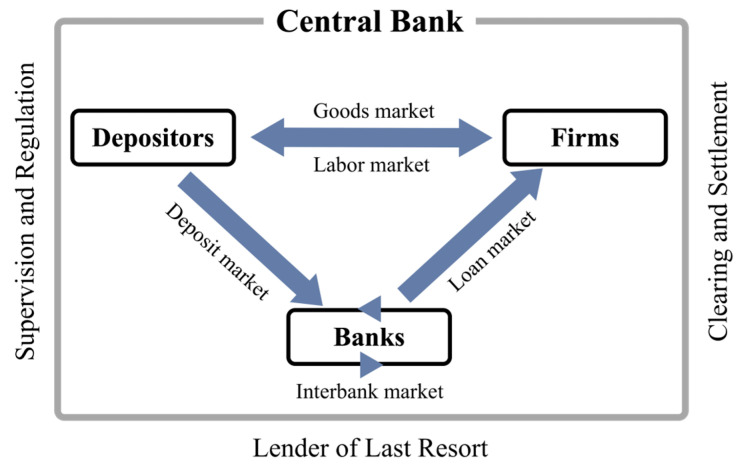
Multi-agent financial network and agent interactions.

Banks collect deposits and extend loans to firms, earning profits from interest-rate spreads. Firms pay wages to hire labor and generate operating revenue from goods sales. These activities take place within a regulatory environment overseen by the central bank, which also provides clearing and settlement services and serves as the lender of last resort. Within this framework, banks are exposed to two fundamental risks: credit risk and liquidity risk. Credit risk arises from potential firm defaults amid market fluctuations, which can generate capital losses for lenders, while liquidity risk reflects funding pressures, such as deposit withdrawals or external shocks, which are often amplified by credit losses. Effective identification and management of these interlinked risks are vital to prevent severe losses, insolvency, and closure. As shown in Figure 1, this paper develops an agent-based financial network model that encompasses banks, depositors, firms, and the central bank, as well as the interactions among these agents in the goods, labor, deposit, loan, and interbank markets. The model is used to analyze resolution strategies under concurrent liquidity shortfalls and credit losses, and to assess how these strategies affect the overall stability of the banking system. For conceptual clarity, the framework is not intended as a strict multiplex network with explicitly separated layers and formal interlayer adjacency structures. Rather, it is designed as a multi-agent financial network in which liquidity and credit risks propagate through coupled channels associated with the asset and liability sides of banks’ balance sheets.

### 2.1. Firms

A firm’s investment directly determines its output. The output of firm i at time t is specified as follows:

(1) $$Y_{i,t}=\phi K_{i,t}$$

where ϕ is the firm’s productivity parameter, and K is its total production cost. The firm finances production through two sources: internal funding, derived from net assets and denoted by $E_{i,t}^{f}$, and external funding, consisting of bank loans and denoted by $L_{i.t}^{f}$. Thus, the firm’s leverage is defined as li,tf=Li,tfEi,tf. The total production inputs for firm i at time t are denoted by $K_{i,t}=E_{i,t}^{f}+L_{i,t}^{f}$.

The unit value of the product varies with market competition. The unit value of firm i’s product is denoted by $u_{i,t}\in \left(0,2\right)$, with $E\left(u_{i,t}\right)=1$. Thus, the sales of firm i are denoted by $S_{i,t}=u_{i,t}Y_{i,t}$. Considering the firm’s financing and labor costs and following the assumptions of Tedeschi et al. [50], the operating profit of firm i is given by:

(2) $$\pi_{i,t}^{f}=S_{i,t}-gr_{i,t}^{f}K_{i,t}$$

where $r_{i,t}^{f}$ denotes the loan interest rate for firm i at time t, and g represents the firm’s comprehensive cost parameter. In the next stage, the firm evaluates its capital demand based on its current capital position and prevailing interest rates.

Considering the marginal exit cost, the capital demand of firm i at time t is expressed as follows:

(3) $$K_{i,t}^{*}=\frac{\phi -gr_{i,t}^{f}}{c\phi gr_{i,t}^{f}}+\frac{E_{i,t-1}^{f}}{2gr_{i,t}^{f}}$$

where c is a parameter related to the firm’s bankruptcy cost. Thus, the loan demand of firm i is expressed as follows:

(4) $$L_{i,t}^{f*}=L_{i,t-1}^{f}-\pi_{i,t-1}^{f}+K_{i,t}^{*}-K_{i,t-1}$$

### 2.2. Banks

Banks collect deposits and extend loans to firms. Accordingly, the balance sheet of bank j is represented as $L_{j,t}^{bf}+L_{j,t}^{bb}+C_{j,t}=E_{j,t}^{b}+B_{j,t}^{bb}+B_{j,t}^{bc}+D_{j,t}$, with the left-hand side representing assets and comprising, in the following order, loans to firms, interbank loans, and cash. The right-hand side represents liabilities and includes net assets, interbank borrowings, central bank borrowings, and deposits, listed in the order shown. Under Basel III, the minimum capital adequacy ratio constrains banks’ lending capacity by limiting excessive balance-sheet expansion. Let $\omega_{0}$ denote the minimum capital adequacy ratio. The credit supply of bank j is given by:

(5) $$CS_{j,t}=\min\left(E_{j,t}^{b}/\omega_{0}-L_{j,t}^{bf}-L_{j,t}^{bb},C_{j,t}\right)$$

Banks extend credit to borrowers according to their risk profiles. According to Grilli et al. [53], the probability that bank j extends credit to a borrower is specified as:

(6) $$p_{i,j,t}=1-\chi_{i,t}{\left(\frac{L_{i,t}}{S_{j,t}}\right)}^{\psi }$$

where ψ is the elasticity parameter, while χ denotes the borrower’s operational risk parameter. As specified in Equation (6), borrowers with higher current indebtedness and operational risk face a lower probability of obtaining bank loans. In contrast, stronger bank credit supply increases the likelihood of loan approval. Borrowers typically choose banks offering the most competitive interest rates among potential lenders. In both the bank–firm credit market and the interbank lending market, bank behavior is governed by the constraints formalized in Equation (6).

When a bank extends credit to a borrower, the loan interest rate is set relative to the central bank’s benchmark rate, with an additional margin applied. The magnitude of this adjustment depends on the bank’s net assets and the borrower’s leverage. Specifically, higher borrower leverage results in a larger premium over the benchmark rate, while stronger bank capitalization reduces the premium. Following Gatti et al. [54], the additional margin applied by bank j above the central bank’s benchmark rate is expressed as follows:

(7) $$f_{i,j,t}=\eta {\left(l_{i,t}\right)}^{\eta }+\eta {\left(E_{j,t}^{b}\right)}^{-\eta }$$

where η denotes the constant parameter governing the loan interest-rate adjustment.

The profit of bank j is given by:

(8) $$\pi_{j,t}^{b}=\sum r_{i,t}^{f}L_{i,j,t}^{bf}+\sum r_{k,t}^{b}L_{k,j,t}^{bb}-\sum r_{j,t}^{b}B_{j,p,t}^{bb}-r_{t}^{c}B_{j,t}^{bc}-r_{t}^{d}D_{j,t}$$

The right side of Equation (8) comprises, in the following order, interest income from firm loans, interest income from interbank loans, interest expenses on interbank borrowings, central bank borrowings, and deposits. $r_{t}^{f},r_{t}^{b},r_{t}^{c},r_{t}^{d}$ denote, respectively, the interest rates on firm loans, interbank borrowings, central bank borrowings, and deposits, subject to the condition $r_{t}^{f}>r_{t}^{c}>r_{t}^{b}>r_{t}^{d}$. Consequently, the net assets of bank j at time t are updated as follows:

(9) $$E_{j,t}^{b}=E_{j,t-1}^{b}+\pi_{j,t}^{b}-Loss_{j,t}$$

where $Loss_{j,t}$ represents the non-performing loan loss incurred by bank j.

In this study, capital adequacy is measured as the ratio of a bank’s net assets to its total risk exposure, which comprises both firm loans ($L_{j,t}^{bf}$) and interbank loans ($L_{j,t}^{bb}$). At time t, the capital adequacy ratio of bank j is updated as follows:

(10) $$\omega_{j,t}=\frac{E_{j,t}^{b}}{L_{j,t}^{bf}+L_{j,t}^{bb}}$$

The cumulative return on capital for bank j is calculated as follows:

(11) $$ROE_{j,t}=\frac{E_{j,t}^{b}-E_{j,0}^{b}}{E_{j,0}^{b}}$$

The expected return on capital for the overall banking system is calculated as follows:

(12) $$EROE_{t}={\bar{ROE}}_{t}*P_{t}$$

where $\bar{ROE}$ represents the average return on capital across the banking system, and P denotes the bank survival probability.

### 2.3. Depositors

Deposits constitute a primary source of bank funding. Following Adão et al. [52], we treat depositors as adaptive agents in the financial network. They monitor banks’ risk conditions on an ongoing basis, and when concerns arise about a bank’s solvency, they withdraw deposits to avoid potential losses. Two types of depositors are distinguished: patient and impatient depositors. In this model, solvency is evaluated based on the bank’s capital adequacy ratio. To focus on the mechanisms through which bank resolution strategies affect systemic stability, the model abstracts from factors such as expectation formation, information asymmetry, or bank opacity. Depositors are instead assumed to have timely access to the information needed for withdrawal decisions and to respond accordingly. Although this assumption may accelerate bank runs and amplify short-term liquidity stress, it removes the confounding role of information frictions and allows the effects of alternative resolution strategies on financial stability to be identified more clearly.

At time t, if the capital adequacy ratio of bank j satisfies the condition $\omega_{j,t}<\omega_{j,t-1}$, impatient depositors perceive elevated operational risk and withdraw their deposits immediately. Patient depositors, on the other hand, withdraw their deposits only with a certain probability, denoted by $P_{W}$. Withdrawn deposits are then redeposited with other banks within the system so that they continue to earn interest.

### 2.4. Risk Resolution Strategies

Given the behavioral rules of the agents described above, we specify a set of bank resolution strategies that are triggered in response to risk shocks within the financial network. At the end of each cycle, banks evaluate firms’ solvency to manage credit risk. When the condition $E_{i,t-1}^{f}+\pi_{i,t}^{f}>0$ is satisfied, firms are deemed solvent, and credit activities proceed according to the agreed terms. Conversely, if $E_{i,t-1}^{f}+\pi_{i,t}^{f}\leq 0$, firms are classified as insolvent. Without any countermeasures, firm defaults may lead to bankruptcy and credit losses through bank-firm linkages. In this model, banks adopt proactive resolution measures to mitigate potential losses. This paper examines two strategies available to banks during credit default shocks:

1. Debt-extension strategy: The bank may roll over a loan maturing at time t to t+1 and classify it as a “loan of concern”, with interest continuing to accrue. During time t+1, the borrower remains under special monitoring and is barred from any additional credit activity. At the end of time t+1, the bank reassesses solvency: if solvency is adequate, the firm’s credit rating is restored and normal production and operations resume; otherwise, the loan is rolled over for one more cycle. Rollovers are allowed up to σ times; if the firm still fails to meet its maturing obligations thereafter, the bank initiates bankruptcy liquidation and recognizes the corresponding credit losses.
2. Debt-relief strategy: The bank applies a preferential interest margin γ and recomputes the interest due from borrowing firms. Under the preferential rate, a firm that can repay at maturity continues production and operations; if solvency remains inadequate, the bank proceeds with bankruptcy liquidation and incurs the corresponding credit losses. In this model, the liquidated firm is replaced by a new entrant with the same initial attributes.

Following the implementation of the above risk resolution strategies, a bank’s capital adequacy ratio fluctuates over time, rising with profitability and falling with credit losses. When the current ratio drops below the previous-period level, impatient depositors may withdraw funds early, triggering liquidity risk. This study considers two strategies available to banks under liquidity stress:

1. Liability-expansion strategy: Banks can enhance liquidity by borrowing in the interbank lending market. If interbank funding falls short, they may seek central bank support at an interest cost. Borrowing volume depends on liquidity needs, while this approach strengthens interbank linkages and may transmit risk across the system.
2. Asset-sale strategy: Banks may liquidate portions of their loan portfolios to reduce exposure to risky assets, prioritizing “loans of concern”. These loans are assumed to depreciate by a percentage θ during the sale. This strategy helps banks satisfy regulatory requirements, albeit at the expense of expected future earnings.

### 2.5. Entropy-Based Measures of Systemic Stability

In this study, the systemic outcomes of different risk resolution strategies are assessed using two indicators: the default probability of the banking system and its expected return. To better characterize the complexity and structural properties of the banking system’s state dynamics, we further introduce a Shannon entropy-based measure of bank states. Specifically, in each simulation period, banks are categorized into three states: normal, liquidity distress, and default. Let the proportion of banks in state k at time t be denoted by $P_{k,t}^{b}$. The bank state entropy is then defined as:

(13) $$H_{t}=-\sum_{k=1}^{3}P_{k,t}^{b}\ln P_{k,t}^{b}$$

The normalized form of the entropy measure is given by:

(14) $${\tilde{H}}_{t}=H_{t}/\ln3$$

State entropy measures the dispersion of bank states across the system and captures the uncertainty associated with their distribution. Importantly, lower entropy should not be interpreted mechanically as evidence of greater systemic stability. It may reflect either a stable state, in which most banks remain normal, or a collapsed state, in which most banks have defaulted. By contrast, higher state entropy indicates a more dispersed and heterogeneous distribution of bank states, with the system occupying a more complex and uncertain mixed state. In particular, when ${\tilde{H}}_{t}=1$, entropy reaches its maximum value: normal, liquidity-distressed, and defaulted banks each account for one-third of the banking system. Thus, bank state entropy is not a direct measure of systemic stability. Rather, it captures the heterogeneity, diversity, and uncertainty of banks’ states within the system. Accordingly, state entropy is used together with default probabilities and expected returns to trace the evolution of systemic stability under different bank resolution strategies.

This study develops a multi-agent financial network model that captures the behavior of heterogeneous financial agents and their interactions within a system exposed to multiple sources of risk. The model provides a tractable framework for simulating financial network dynamics and assessing the systemic effects of alternative bank risk resolution strategies. To maintain analytical tractability, we simplify the representation of banks and firms and abstract from information asymmetries among agents. These assumptions allow the analysis to focus on bank–firm credit linkages and on liquidity stress induced by depositor withdrawals, thereby providing a clearer assessment of how alternative resolution strategies shape financial network stability.

**Figure 2:**
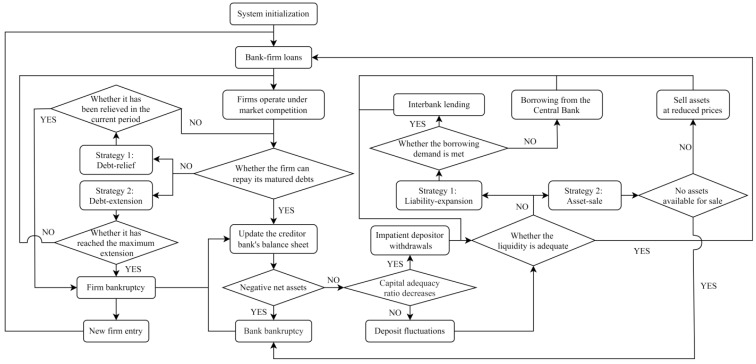
The dynamic evolution of the financial network system.

Figure 2 depicts the dynamic evolution of the financial network system, driven by agent behaviors and banks’ resolution responses to risk shocks. The figure summarizes how credit and liquidity shocks propagate through the model and how banks respond at each decision point. When a firm defaults, the affected bank selects one of the two credit risk resolution strategies. When depositor withdrawals generate liquidity stress, the bank implements one of the two liquidity risk resolution strategies. A bank may be liquidated if losses are substantial or if liquidity requirements are unmet.

## 3. Results and Discussion

### 3.1. Simulation Settings

The model parameters are specified as follows. The system comprises $N_{B}=50$ banks, $N_{F}=500$ firms, and $N_{H}=5000$ depositors. Firms’ initial net assets are set at $E_{0}^{f}=35$, and banks extend an initial loan of $L_{0}^{f}=65$ to each firm. Banks’ initial cash and credit supply are given by $C_{0}=CS_{0}=E_{0}^{b}$. Initial interbank borrowing and central bank borrowing are set to zero to rule out the influence of pre-existing liquidity assistance or interbank liability exposures in the baseline setting. Several financial parameters are calibrated to reflect key features of China’s banking sector. Data from the People’s Bank of China show that, since 2019, the core Tier 1 capital adequacy ratio of Chinese commercial banks has remained broadly within the range of 10.5–11%. The one-year LPR has been approximately 3–4.25%, and the one-year deposit rate has been around 1.5%. Accordingly, the initial bank capital adequacy ratio is set to $\omega_{0}=0.1$, and initial interest rates are set within the range of 2–4.5%.

The firm-level parameters are primarily based on Tedeschi et al. [50]. The comprehensive cost parameter is set to g=1.2, the bankruptcy cost parameter to c=1, and firm productivity to ϕ=0.05. With these values, most firms are close to break-even at the start of the simulation. This initialization limits the role of ex ante firm heterogeneity and helps isolate the effects of risk shocks, credit contraction, and liquidity stress within the financial network. Following Grilli et al. [53], the elasticity parameter is set to ψ=0.1. Because firms are more directly exposed to production and operating uncertainty than banks, the operational risk parameters are set to 0.7 for firms and 0.3 for banks. Depositor behavior follows Adão et al. [52]. Depositors are evenly divided into patient and impatient types, with each group accounting for 50% of the depositor population. Impatient depositors withdraw immediately, whereas patient depositors withdraw with probability $P_{W}=0.1$. All parameter values are summarized in Table 1.

The simulation experiments are designed around a set of predefined risk resolution strategies. We first compare the systemic stability outcomes of two liquidity risk resolution strategies: the liability-expansion strategy and the asset-sale strategy. We then assess the effects of the debt-extension strategy and the debt-relief strategy under each liquidity risk resolution scenario. Each simulation run lasts for up to 300 time steps and terminates earlier if all banks fail. To reduce the influence of stochastic variation, each experiment is repeated 100 times, and the results reported below are based on these repeated runs.

### 3.2. Liquidity Risk Resolution

**Figure 3:**
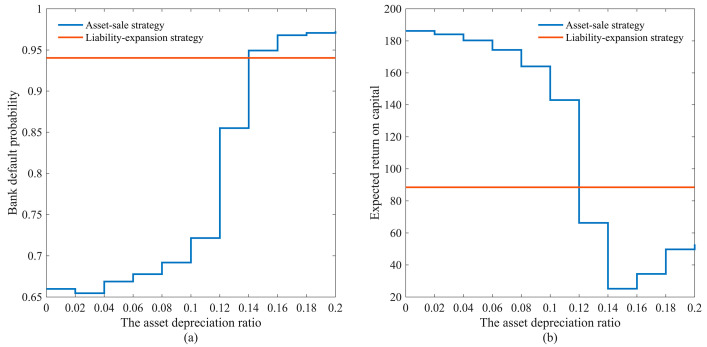
Effects of different liquidity risk resolution strategies on banking system stability. (**a**) Bank default probability. (**b**) Banks’ expected return on capital. (Note: Under the liability-expansion strategy, banks’ borrowing needs are consistently met with central bank support).

**Figure 4:**
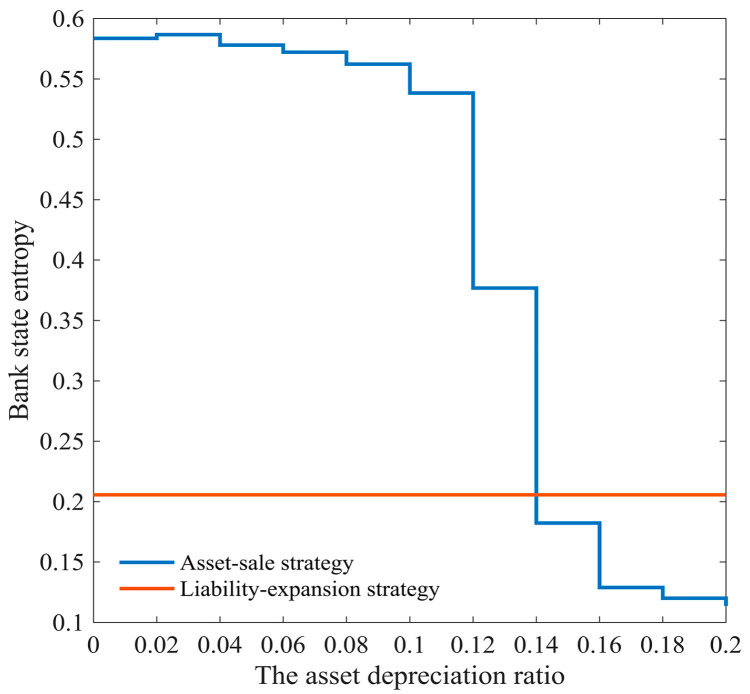
Bank state entropy under different liquidity risk resolution strategies.

This study examines how two liquidity risk resolution strategies—the liability-expansion and asset-sale strategies—affect the stability of the banking system in a dynamic financial network subject to liquidity risk. The results are shown in Figure 3. The results indicate that the effectiveness of the asset-sale strategy is closely related to the extent of asset depreciation. As depreciation increases, both the default probability and the expected return on capital first decline and then rise. When depreciation remains relatively low, banks adopting the asset-sale strategy exhibit lower default probabilities and higher expected returns on capital than those pursuing the liability-expansion strategy. The distribution of bank states further supports this finding (Figure 4). Under the liability-expansion strategy, banks’ state entropy remains persistently low. When considered alongside the default probability, this pattern suggests that the system becomes increasingly concentrated in the default state. By contrast, under the asset-sale strategy, state entropy remains higher than under the liability-expansion strategy when asset depreciation is limited. This implies that moderate asset depreciation, defined in our simulations as a depreciation ratio below 0.12, helps preserve heterogeneity within the banking system, thereby mitigating excessive risk concentration and reducing the likelihood of systemic collapse.

**Figure 5:**
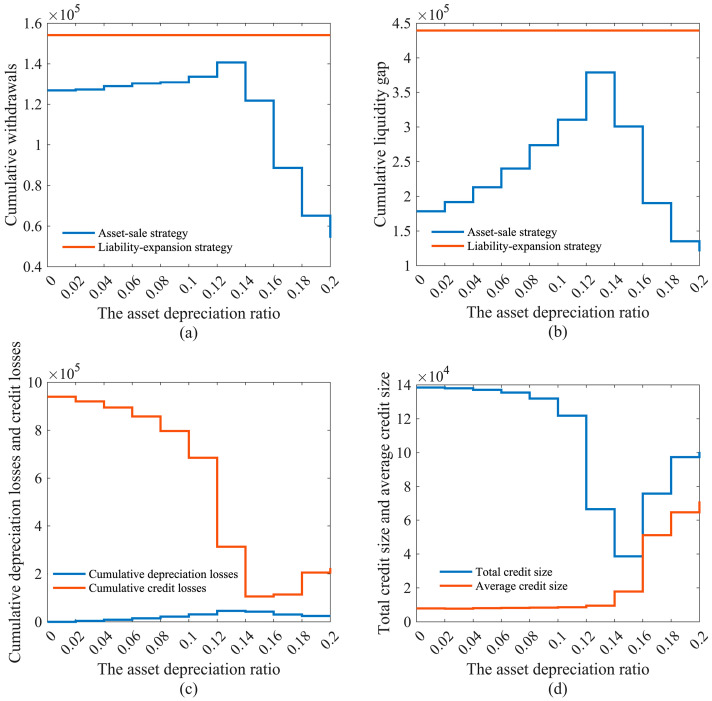
Performance of the financial network system under the asset-sale strategy. (**a**) Depositors’ cumulative withdrawals under different liquidity risk resolution strategies. (**b**) Banks’ cumulative liquidity gap under different liquidity risk resolution strategies. (**c**) Cumulative depreciation losses from asset sales and cumulative credit losses from firm defaults under the asset-sale strategy. (**d**) Banks’ total credit size and average credit size under the asset-sale strategy.

The analysis shows that when banks adopt the liability-expansion strategy, they cover liquidity shortfalls by borrowing in the interbank market and from the central bank, thereby increasing leverage and funding-cost pressures. As profitability declines, depositors withdraw funds, which further exacerbates liquidity stress. As illustrated in Figure 5a,b, both cumulative withdrawals and the liquidity gap are significantly larger under the liability-expansion strategy than under the asset-sale strategy. The resulting liquidity pressure increases banks’ exposure to default risk. In contrast, when banks sell assets during liquidity crises, they reduce overall credit exposure while realizing losses on principal and earnings. The effectiveness of the asset-sale strategy in stabilizing the banking system, therefore, depends critically on the depreciation ratio applied to asset sales.

At lower depreciation levels, principal losses are modest relative to credit default risks. As depreciation increases, banks must liquidate more credit assets to meet liquidity needs, thereby reducing total bank-firm credit and lowering default probability. Meanwhile, depreciation losses increase (Figure 5c), which lowers return on capital, although returns remain higher under the asset-sale strategy than under the liability-expansion strategy. Once depreciation exceeds the critical range of 0.12–0.14 in our simulations, liquidity gaps widen, asset-sale losses intensify, and the advantage of the asset-sale strategy diminishes. This indicates that, beyond this range, as depreciation increases, the impairment losses from asset sales exceed the incremental cost of liability expansion, reversing the relative performance of the two liquidity risk resolution strategies. Further asset depreciation reduces system returns and raises default probability. Surviving banks that retain remaining assets experience an increase in average credit size (Figure 5d), leading to upward adjustments in expected returns.

Overall, the liability-expansion strategy increases banks’ financing costs, reduces profitability, and prompts further withdrawals. Although it may temporarily alleviate liquidity strain, its longer-term effect on bank performance is detrimental. By contrast, the effectiveness of the asset-sale strategy depends on the degree of asset depreciation: when depreciation is low, the asset-sale strategy outperforms the liability-expansion strategy; when depreciation is high, its effectiveness declines. Banks should therefore adapt their liquidity risk resolution strategies to market conditions to balance stability and profitability.

### 3.3. Credit Risk Resolution

When firms fail to repay maturing principal or interest on their loans, banks face credit default risk. Whereas Section 3.2 focused on liquidity risk resolution, it did not consider credit default exposures associated with debtor firms in the financial network. To mitigate such losses, banks employ measures designed to prevent defaults or reduce default-related losses. This section examines two resolution strategies, the debt-extension strategy and the debt-relief strategy, that banks may adopt when debtors struggle to meet their repayment obligations, and discusses their implications for the stability of the banking system as a whole.

#### 3.3.1. Debt-Extension Strategy

**Figure 6:**
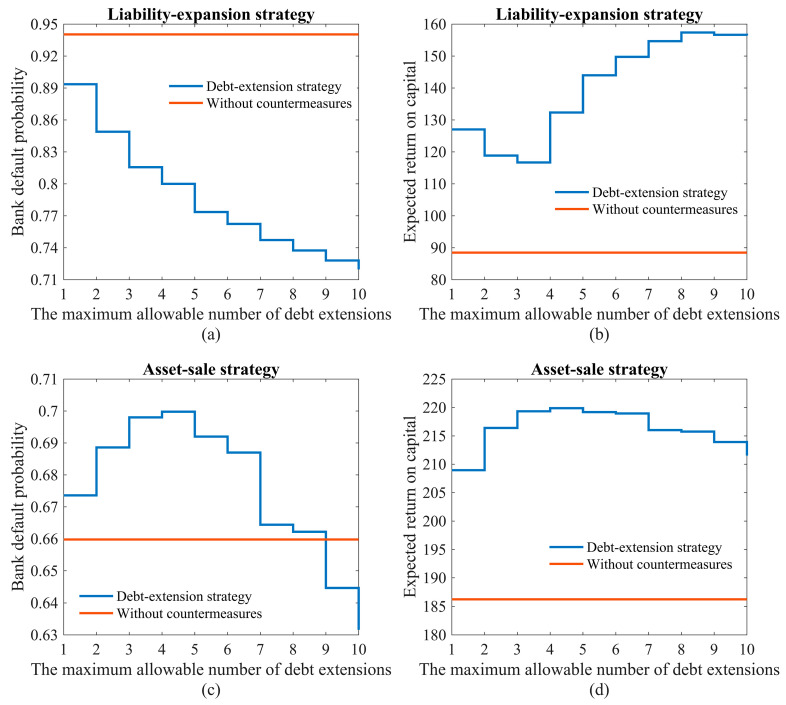
Effects of bank debt extension on banking system stability under different liquidity risk resolution strategies. (**a**) Bank default probability under the liability-expansion strategy. (**b**) Banks’ expected return on capital under the liability-expansion strategy. (**c**) Bank default probability under the asset-sale strategy. (**d**) Banks’ expected return on capital under the asset-sale strategy. (θ=0).

**Figure 7:**
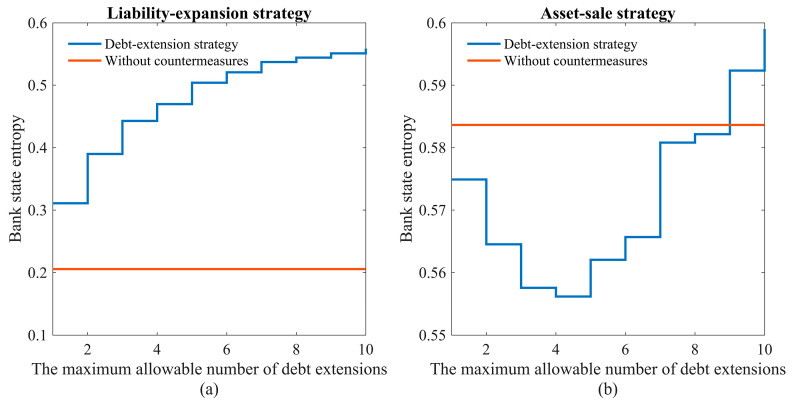
Bank state entropy under bank debt extension across different liquidity risk resolution strategies. (**a**) Bank state entropy under the liability-expansion strategy. (**b**) Bank state entropy under the asset-sale strategy.

This study examines the effect of banks’ adoption of the debt-extension strategy on systemic stability by allowing the maximum allowable number of debt extensions (σ) to vary from 1 to 10. As shown in Figure 6 and Figure 7, the effects vary markedly with the liquidity risk resolution strategy used.

Under the liability-expansion strategy, the system’s default probability declines sharply as the maximum allowable number of debt extensions increases, while the expected return on capital follows a “fall–rise–fall” pattern. When confronted with credit default shocks, banks that grant maturity extensions to debtor firms substantially lower systemic default risk and improve capital returns compared with banks that take no countermeasures. Figure 7a shows that, as the maximum allowable number of debt extensions increases, the system-wide default probability declines, whereas bank state entropy rises. The increase in entropy reflects greater cross-bank heterogeneity, suggesting that a higher extension limit is associated with greater risk dispersion in the banking system and a more stable financial network structure.

In contrast, under the asset-sale strategy, both the default probability and the expected return initially increase and then decline as the maximum allowable number of debt extensions increases. At moderate levels, debt extension improves returns but has limited effect in reducing systemic risk. Figure 7b further indicates that, under the asset-sale strategy, debt extension tends to intensify risk contagion, reduce heterogeneity in banks’ state distributions, and undermine the structural stability of the financial network. Taken together with the bank default probabilities and expected returns reported in Figure 6c,d, the results suggest that only relatively high debt-extension thresholds, above 8 in our simulations, can meaningfully contain default risk.

**Figure 8:**
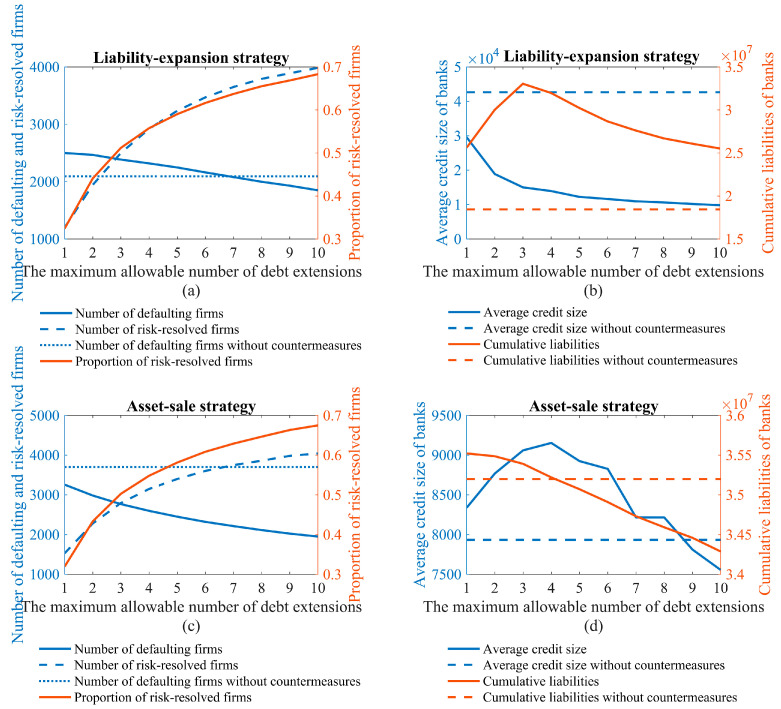
Financial network outcomes after debt extension under different liquidity risk resolution strategies. (**a**) Number of defaulting firms (left axis), number of risk-resolved firms (left axis), and proportion of risk-resolved firms (right axis) under the liability-expansion strategy. (**b**) Banks’ average credit size (left axis) and cumulative liabilities (right axis) under the liability-expansion strategy. (**c**) Number of defaulting firms (left axis), number of risk-resolved firms (left axis), and proportion of risk-resolved firms (right axis) under the asset-sale strategy. (**d**) Banks’ average credit size (left axis) and cumulative liabilities (right axis) under the asset-sale strategy.

Figure 8 further shows that, regardless of the liquidity risk resolution strategy, the number of defaulting firms declines, while the proportion of firms whose default risk is resolved increases as the maximum allowable number of debt extensions increases (Figure 8a,c). Debt extension significantly improves firms’ survival prospects and reduces bankruptcy risk. When defaults occur, banks prevent immediate liquidation by extending debt maturities; firms that return to profitability in subsequent cycles can repay their obligations, helping banks recover principal and interest and avoid credit losses. A greater debt-extension limit provides firms with more time to restore solvency, thereby further improving the probability of successful resolution.

Under the liability-expansion strategy, larger asset positions are associated with higher leverage and funding costs. Debt extension lowers bankruptcy risk and enables banks to recover more principal and interest, thereby reducing the default probability. However, this also prolongs banks’ capital recovery process. When the allowable number of debt extensions is small**,** the proportion of firms whose default risk is resolved remains low, while banks’ liabilities are elevated and funding costs rise (Figure 8b), driving returns downward. As the maximum allowable number of debt extensions increases, the probability of successful resolution and recovery rates improve, boosting returns. However, once risks are resolved, firms may curtail borrowing due to weakened balance sheets, reducing average loan sizes and returns. An excessively high debt-extension limit therefore lowers expected returns again.

Under the asset-sale strategy, banks liquidate loan assets and waive unpaid interest to obtain liquidity immediately. This reduces risk by contracting lending activity, lowering credit exposure, and accelerating cash recovery. By contrast, debt extension eases firms’ repayment pressure by lengthening loan maturities. For banks, however, it leaves credit exposure largely intact while postponing principal recovery and slowing asset-side cash inflows. These mechanisms therefore operate in opposite directions. As shown in Figure 8d, under the asset-sale strategy, debt extension markedly increases banks’ average credit size, thereby offsetting the exposure-reducing effect of asset sales. As the maximum allowable number of debt extensions increases, this offset becomes more pronounced, which increases banks’ default probability. However, because debt extension reduces the number of defaulting firms, the return on capital increases. As the maximum allowable number of debt extensions continues to increase, reduced loan demand lowers banks’ risk exposure, gradually decreasing bank default probability but also constraining returns. Once the maximum allowable number of debt extensions exceeds a certain threshold, firm default risk drops markedly, alleviating credit losses; system-wide defaults then fall below the levels observed when no countermeasures are adopted.

Overall, the debt-extension strategy helps ease firms’ repayment burdens and strengthen their resilience to credit stress. A higher allowable number of extensions increases the likelihood of distress resolution and promotes system stability. Nevertheless, this strategy partly offsets the asset-sale strategy, creating a trade-off in their effects. Hence, the debt-extension strategy is most effective when banks manage liquidity through liability expansion rather than asset sales.

#### 3.3.2. Debt-Relief Strategy

**Figure 9:**
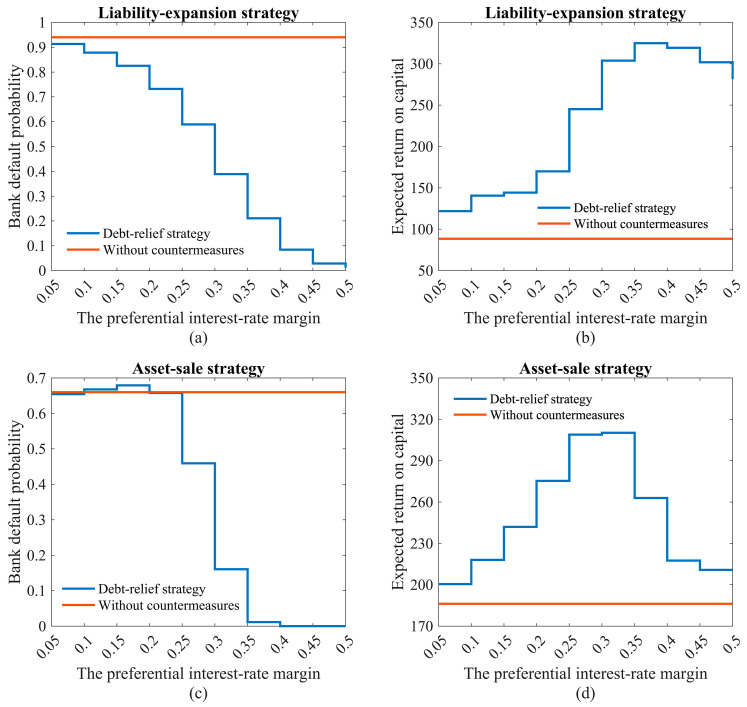
Effects of bank debt relief on banking system stability under different liquidity risk resolution strategies. (**a**) Bank default probability under the liability-expansion strategy. (**b**) Banks’ expected return on capital under the liability-expansion strategy. (**c**) Bank default probability under the asset-sale strategy. (**d**) Banks’ expected return on capital under the asset-sale strategy (θ=0).

**Figure 10:**
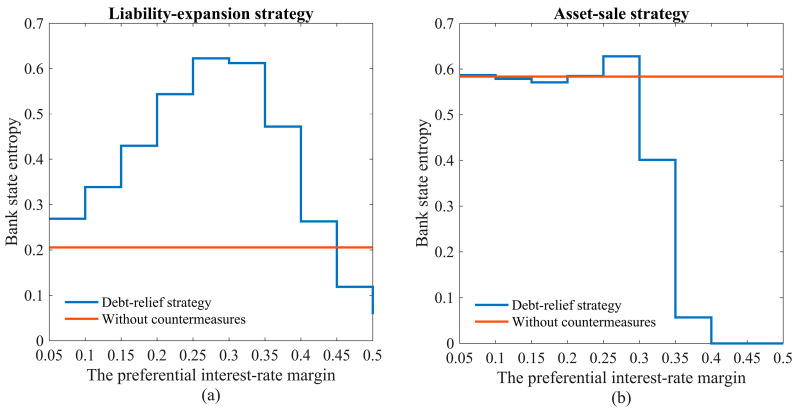
Bank state entropy under bank debt relief across different liquidity risk resolution strategies. (**a**) Bank state entropy under the liability-expansion strategy. (**b**) Bank state entropy under the asset-sale strategy.

To examine the effects of the debt-relief strategy on the stability of the banking system, we vary the preferential interest-rate margin, γ, from 0.05 to 0.50 in increments of 0.05. At γ=0.5, banks’ default probability reaches zero or a level very close to zero. The results are reported in Figure 9 and Figure 10. Across liquidity management regimes, the results show consistent patterns.

Under the liability-expansion strategy, systemic default probability declines monotonically as the preferential interest-rate margin increases, while the expected return on capital initially rises and then falls. Compared with the case in which no countermeasures are taken, debt relief materially lowers default risk and yields higher returns. Meanwhile, bank state entropy first rises and then falls (Figure 10a). Taken together with bank default probability, this pattern indicates that the banking system transitions from a collapsed state, in which most banks have defaulted, to a stable state, in which most banks remain in the normal state.

Under the asset-sale strategy, both default probability and expected return on capital initially increase and then decline as the preferential interest-rate margin increases. At a low preferential margin, default risk exceeds that under the no-countermeasure benchmark; at a higher preferential margin, risk falls substantially, but returns decline, while remaining above those under the no-countermeasure benchmark. Figure 10b shows that a low preferential margin has only a limited effect on the distribution of bank states: risk transmission remains limited in scope, with little observable improvement. By contrast, a higher preferential margin shifts banks toward the normal state, thereby lowering banks’ default probability and substantially enhancing overall systemic stability.

**Figure 11:**
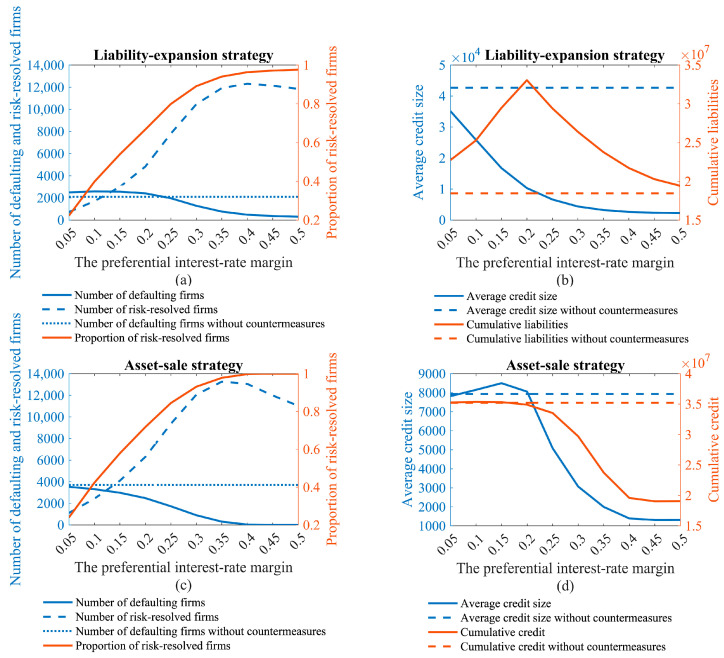
Financial network outcomes after debt relief under different liquidity risk resolution strategies. (**a**) Number of defaulting firms (left axis), number of risk-resolved firms (left axis), and proportion of risk-resolved firms (right axis) under the liability-expansion strategy. (**b**) Banks’ average credit size (left axis) and cumulative liabilities (right axis) under the liability-expansion strategy. (**c**) Number of defaulting firms (left axis), number of risk-resolved firms (left axis), and proportion of risk-resolved firms (right axis) under the asset-sale strategy. (**d**) Banks’ average credit size (left axis) and cumulative credit (right axis) under the asset-sale strategy.

Figure 11 shows that debt relief substantially reduces firm credit defaults, with the proportion of firms whose risk is resolved increasing (Figure 11a,c). Preferential interest rates directly ease repayment pressure: if relief fails, losses are no greater than under immediate liquidation; if it succeeds, defaults are avoided, and only interest concessions are incurred. A larger preferential margin thus raises the likelihood of timely repayment and lowers banks’ exposure to credit default. Notably, when liquidity is managed via an asset-sale strategy, systemic default probability can exceed that under the no-countermeasure benchmark at a low preferential margin (below 0.15 in our simulations).

Under the asset-sale strategy, banks reduce the size of their credit portfolios, which leads to a decline in interest income. At a low preferential margin, residual default risk remains high, and concessions slightly increase systemic default probability. At a higher preferential margin, sharp reductions in default risk and credit losses lower systemic defaults; when the preferential margin reaches 0.4, firm defaults fall to zero, and system-wide defaults also decline to zero. This decline is more pronounced than under the liability-expansion strategy.

Banks’ voluntary interest concessions mitigate credit default risk, preserve residual interest income, and increase expected returns. As more firms resolve distress, they reduce borrowing because of weakened balance sheets and lower loan demand, causing returns to decline after peaking. When combined with asset sales, deliberate balance sheet contraction brings forward the post-peak decline in returns and makes it steeper than that observed under the liability-expansion strategy.

In sum, the debt-relief strategy eases borrowers’ debt burdens and lowers credit default risk; a higher preferential margin increases the likelihood of timely repayment. Across both liquidity strategies, debt relief can mitigate systemic risk and improve profitability, although large concessions may erode equity. Combining the debt-relief strategy with the asset-sale strategy can generate substantial stability gains, but very low preferential margins may be counterproductive, as they can increase default risk. Banks should therefore calibrate preferential interest rates to their risk appetite and chosen liquidity management strategy, based on rigorous risk assessment.

### 3.4. Robustness Analysis

This study examines how banks’ risk resolution strategies affect the stability of the banking system under coupled liquidity and credit risks, with particular attention to the interactions among different resolution strategies. The model developed in Section 2 implies that firms’ initial net assets, initial loan size, and adjustments to bank loan rates directly shape firms’ capacity to absorb shocks. These firm-level conditions, in turn, affect the risk exposure of the financial network and have implications for the stability of the banking system.

**Figure 12:**
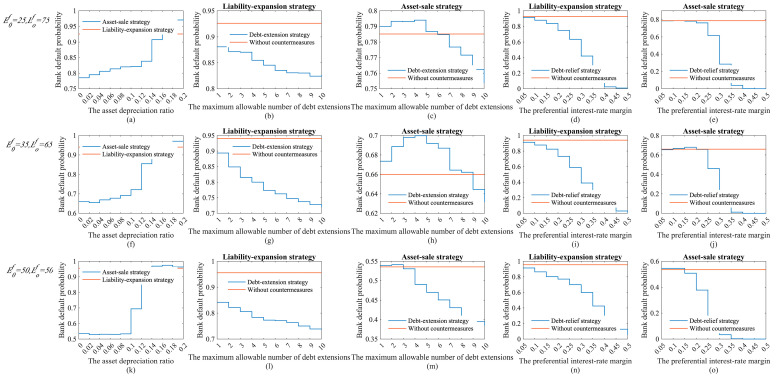
Default probability of the banking system under alternative settings for firms’ initial net assets and loans. (Panels (**a**,**f**,**k**): Bank default probability when banks adopt different liquidity risk resolution strategies under alternative settings for firms’ net assets and loans. Panels (**b**,**g**,**l**): Bank default probability when banks adopt the liability-expansion strategy together with the debt-extension strategy under alternative settings for firms’ net assets and loans. Panels (**c**,**h**,**m**): Bank default probability when banks adopt the asset-sale strategy together with the debt-extension strategy under alternative settings for firms’ net assets and loans. Panels (**d**,**i,n**): Bank default probability when banks adopt the liability-expansion strategy together with the debt-relief strategy under alternative settings for firms’ net assets and loans. Panels (**e**,**j**,**o**): Bank default probability when banks adopt the asset-sale strategy together with the debt-relief strategy under alternative settings for firms’ net assets and loans).

**Figure 13:**
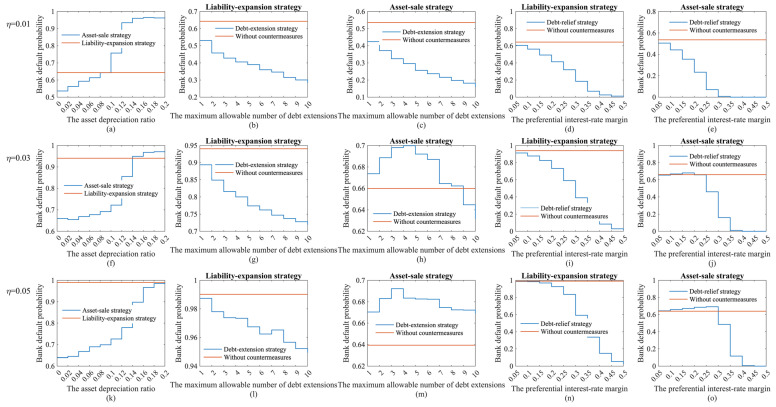
Default probability of the banking system under alternative bank loan rate adjustment parameters. (Panels (**a**,**f**,**k**): Bank default probability when banks adopt different liquidity risk resolution strategies under alternative bank loan rate adjustment parameters. Panels (**b**,**g**,**l**): Bank default probability when banks adopt the liability-expansion strategy together with the debt-extension strategy under alternative bank loan rate adjustment parameters. Panels (**c**,**h**,**m**): Bank default probability when banks adopt the asset-sale strategy together with the debt-extension strategy under alternative bank loan rate adjustment parameters. Panels (**d**,**i**,**n**): Bank default probability when banks adopt the liability-expansion strategy together with the debt-relief strategy under alternative bank loan rate adjustment parameters. Panels (**e**,**j**,**o**): Bank default probability when banks adopt the asset-sale strategy together with the debt-relief strategy under alternative bank loan rate adjustment parameters).

To assess the robustness of the simulation results, we vary the key parameter settings used in the baseline experiments. In Section 3.1, the baseline parameters are set to $E_{0}^{f}=35,L_{0}^{f}=65$ and η=0.03. In this section, we compare the baseline results with four alternative settings: $E_{0}^{f}=25,L_{0}^{f}=75$, $E_{0}^{f}=50,L_{0}^{f}=50$, η=0.01 and η=0.05. The results are shown in Figure 12 and Figure 13.

Figure 12 presents the default probability of the banking system under alternative assumptions regarding firms’ initial net assets and initial loan sizes, which jointly determine initial leverage. The results are broadly consistent across the three parameter settings and confirm the main findings from the baseline analysis. When banks are exposed to liquidity risk, larger depreciation of assets upon sale generally reduces the effectiveness of the asset-sale strategy. At moderate depreciation levels, however, the asset-sale strategy performs better than the liability-expansion strategy. Moreover, the critical depreciation threshold increases with firms’ initial leverage. Under credit risk, when banks adopt liability expansion as the liquidity risk resolution strategy, allowing a larger number of debt extensions strengthens the risk-mitigating effect of the debt-extension strategy. This effect, however, becomes weaker as firms’ initial leverage increases. When banks instead rely on asset sales, the results remain consistent with those reported in Section 3.3.1. If the allowable number of debt extensions is low, the debt-extension strategy may increase banks’ default probability. As firms’ initial leverage declines, however, the debt-extension strategy becomes more effective in reducing bank risk. This finding suggests that firms with lower leverage are better able to use the debt-extension strategy to ease repayment pressure and contain default risk. The results for the debt-relief strategy are likewise consistent with the baseline findings. Under liability expansion, larger preferential interest-rate margins reduce banks’ default probability. Under asset sales, small preferential interest-rate margins slightly increase banks’ default probability. Overall, however, the debt-relief strategy becomes more effective as firms’ initial leverage decreases.

Figure 13 shows that, under alternative settings for the bank loan rate adjustment parameter, the default probability of the banking system follows a broadly similar pattern, consistent with the baseline results. As the bank loan rate adjustment parameter rises, however, the effectiveness of the risk resolution strategies generally weakens. Under liquidity risk, the asset-sale strategy outperforms the liability-expansion strategy only when asset depreciation is relatively small. Moreover, this effective range narrows as the bank loan rate adjustment parameter increases.

When banks use debt extension to manage credit risk, the results under the liability-expansion strategy remain fully consistent with the baseline findings. Under the asset-sale strategy, however, the effect depends on the bank loan rate adjustment parameter. When the adjustment is small, firms face relatively mild repayment pressure, and the probability of risk resolution rises as the allowable number of debt extensions increases. When the adjustment becomes larger, the robustness results mirror the baseline findings: the debt-extension strategy and the asset-sale strategy generate offsetting effects over a certain range, thereby increasing banks’ default probability.

A similar pattern emerges for debt relief. When combined with the liability-expansion strategy, debt relief reduces the default probability of the banking system. When combined with the asset-sale strategy, however, the effect again depends on the bank loan rate adjustment. If the adjustment is small, firms’ repayment pressure remains limited, and the two strategies reinforce each other more strongly. By contrast, when the adjustment becomes substantial, the results are consistent with the baseline analysis: small preferential interest-rate margins may increase the default probability of the banking system, and this adverse effect becomes more pronounced as the bank loan rate adjustment parameter rises.

Taken together, although the results differ to some extent across alternative initial conditions, the different risk resolution strategies display broadly consistent patterns. This finding is in line with the evolutionary dynamics of the financial network and provides further support for the robustness of the main findings.

## 4. Conclusions

This study develops a dynamic multi-agent financial network model that captures the interactions among banks, depositors, firms, and the central bank, with liquidity shocks and credit defaults explicitly incorporated. Using this framework, we assess how different bank resolution strategies affect systemic stability under coupled liquidity and credit risks and examine the interplay among these strategies.

When banks face liquidity risk, the liability-expansion strategy sacrifices profitability in order to maintain balance-sheet size and ease liquidity pressures. However, by increasing leverage, it may undermine depositor confidence, widen liquidity gaps, and, in severe cases, trigger bank runs that lead to systemic collapse. The effectiveness of the asset-sale strategy depends on the extent of asset devaluation during sale. When the depreciation ratio is relatively low, at below 0.12 in our simulations, banks can obtain sufficient liquidity while incurring limited valuation losses, thereby easing liquidity stress. A moderate level of asset depreciation also helps preserve heterogeneity within the banking system, thereby preventing excessive risk concentration. Once asset depreciation exceeds a certain threshold, namely, 0.12-0.14 in our experimental setting, the losses banks incur from asset sales become sufficiently large to reverse the relative effectiveness of the two liquidity strategies. This study suggests that, in real-world resolution practice, information asymmetry and market sensitivity make the liability-expansion strategy more suitable for short-term liquidity management when the liquidity gap is modest. For larger liquidity gaps, resolution authorities and bank risk management departments should carefully assess market conditions and adopt flexible resolution strategies for available-for-sale assets in order to secure sufficient liquidity. Such an approach can limit losses from indiscriminate asset sales while preserving the stability of the banking system.

When banks face credit risk, the debt-extension strategy gives borrowers additional time to ease repayment pressure, thereby preventing credit defaults. However, implementing this strategy requires banks to maintain their credit size, which conflicts with the asset-sale strategy used to alleviate liquidity constraints, thereby intensifying risk contagion and disrupting the structure of the financial network. These two strategies, therefore, entail a utility trade-off. The combined use of these two strategies becomes effective in reducing systemic risk only when the maximum allowable number of debt extensions exceeds a sufficiently high threshold, above 8 in our simulations. The debt-extension strategy is particularly suitable for banks seeking to address liquidity risks by expanding liabilities, as it mitigates risk materialization and promotes a more dispersed distribution of risk. In contrast, the essence of the debt-relief strategy lies in banks’ deliberate decision to forgo a portion of profits to prevent greater principal losses, thereby helping to preserve a stable banking structure in which most banks remain in a normal state. Consequently, a higher preferential interest-rate margin corresponds to lower systemic risk but greater pressure on banks’ equity positions. In our simulation setting, an interest-rate reduction of 30–35% appears to be appropriate. These findings suggest that, before formulating preferential interest-rate policies, banks’ risk management departments should conduct comprehensive assessments aligned with their liquidity risk management strategies and establish a balanced risk appetite framework.

This study has several limitations. First, the analysis rests on the simplifying assumption of perfect information. The model therefore abstracts from information frictions, such as expectation formation, information asymmetry, and bank opacity. Depositors are assumed to have access to the information needed to make their decisions, while banks are assumed to manage liquidity and credit risks according to pre-specified resolution strategies. This study does not seek to model agents’ learning under incomplete information; rather; it examines how bank resolution strategies affect systemic stability when liquidity and credit risks are jointly present. Within this scope, the perfect-information assumption provides a tractable framework for isolating the core contagion mechanisms. The robustness checks suggest that parameter choices affect the quantitative magnitudes of the results, but the key conclusions remain unchanged. Overall, the baseline findings are robust. Second, the study relies primarily on numerical simulations, and the results have not yet been validated using real-world data.

Future research could extend the present framework by incorporating information asymmetry and dynamic strategy selection, thereby allowing a more nuanced analysis of risk transmission and the effectiveness of mitigation measures. Future work could also validate the model against bank-level data or introduce macroeconomic shocks to examine how shifts in the macro-financial environment affect systemic stability. The findings of this study offer useful implications for regulatory authorities and bank risk management departments when designing and implementing prudent risk resolution strategies.

## Figures and Tables

**Table 1:** Parameter settings.

Parameter	Description	Value	Parameter	Description	Value
$N_{B}$	Number of banks	50	ψ	Elasticity parameter	0.1
$N_{F}$	Number of firms	500	χ	Operational risk parameter	0.7/0.3
$N_{H}$	Number of depositors	5000	η	Loan rate adjustment parameter	0.03
$E_{0}^{f}$	Initial net assets of firms	35	$P_{W}$	Withdrawal probability	0.1
$L_{0}^{f}$	Initial bank loans to firms	65	$r^{f}$	Firm loan rate	0.045
ϕ	Productivity of firms	0.05	$r^{c}$	Central bank borrowing rate	0.035
g	Comprehensive cost parameter	1.2	$r^{b}$	Interbank borrowing rate	0.025
c	Bankruptcy cost parameter	1	$r^{d}$	Deposits rate	0.02
$\omega_{0}$	Initial capital adequacy ratio	0.1			

## Data Availability

The data are available from the corresponding authors upon request.

## References

[1] Cornett M.M., McNutt J.J., Strahan P.E., Tehranian H. (2011). Liquidity risk management and credit supply in the financial crisis. J. Financ. Econ..

[2] Chen W.D., Chen Y., Huang S.C. (2021). Liquidity risk and bank performance during financial crises. J. Financ. Stab..

[3] Chen Y.P., Guo R.J., Huang R.L. (2009). Two stages credit evaluation in bank loan appraisal. Econ. Model..

[4] Kavussanos M.G., Tsouknidis D.A. (2016). Default risk drivers in shipping bank loans. Transp. Res. Part E-Logist. Transp. Rev..

[5] Nekhili R., Foglia M., Bouri E. (2023). European bank credit risk transmission during the Credit Suisse collapse. Financ. Res. Lett..

[6] Glasserman P., Young B.P. (2015). How likely is contagion in financial networks?. J. Bank. Financ..

[7] Hausenblas V., Kubicová I., Lešanovská J. (2015). Contagion risk in the Czech financial system: A network analysis and simulation approach. Econ. Syst..

[8] Diamond D.W., Rajan R.G. (2011). Fear of fire sales, illiquidity seeking, and credit freezes. Q. J. Econ..

[9] Kesraoui A., Lachaab M., Omri A. (2022). The impact of credit risk and liquidity risk on bank margins during economic fluctuations: Evidence from MENA countries with a dual banking system. Appl. Econ..

[10] Liu J.J., Guo K., Tang F.C., Wang Y., Wang S. (2023). The effect of the disposal of non-performing loans on interbank liquidity risk in China: A cash flow network-based analysis. Q. Rev. Econ. Financ..

[11] Adrian T., Shin H.S. (2009). Money, liquidity, and monetary policy. Am. Econ. Rev..

[12] Ratnovski L., Huang R. (2009). Why are Canadian Banks More Resilient?.

[13] Acharya V.V., Mora N. (2015). A crisis of banks as liquidity providers. J. Financ..

[14] Choudhary M.A., Limodio N. (2022). Liquidity risk and long-term finance: Evidence from a natural experiment. Rev. Econ. Stud..

[15] Iyer R., Peydró J.L., da-Rocha-Lopes S., Schoar A. (2014). Interbank liquidity crunch and the firm credit crunch: Evidence from the 2007–2009 crisis. Rev. Financ. Stud..

[16] Grundke P., Kühn A. (2020). The impact of the Basel III liquidity ratios on banks: Evidence from a simulation study. Q. Rev. Econ. Financ..

[17] Ferrara G., Langfield S., Liu Z.J., Ota T. (2019). Systemic illiquidity in the interbank network. Quant. Financ..

[18] Thakor A.V. (2018). Post-crisis regulatory reform in banking: Address insolvency risk, not illiquidity!. J. Financ. Stab..

[19] Jiménez G., Ongena S., Peydró J.L., Saurina J. (2014). Hazardous times for monetary policy: What do twenty-three million bank loans say about the effects of monetary policy on credit risk-taking?. Econometrica.

[20] Gersl A., Jakubik P., Kowalczyk D., Ongena S., Peydró J.-L. (2015). Monetary conditions and banks’ behaviour in the Czech Republic. Open Econ. Rev..

[21] Bonfim D., Dias D.A., Richmond C. (2012). What happens after corporate default? Stylized facts on access to credit. J. Bank. Financ..

[22] Agostino M., Gagliardi F., Trivieri F. (2012). Bank competition, lending relationships and firm default risk: An investigation of Italian SMEs. Int. Small Bus. J..

[23] Iosifidi M., Kokas S. (2015). Who lends to riskier and lower-profitability firms? Evidence from the syndicated loan market. J. Bank. Financ..

[24] Crawford G.S., Pavanini N., Schivardi F. (2018). Asymmetric information and imperfect competition in lending markets. Am. Econ. Rev..

[25] Betz J., Krüger S., Kellner R., Rösch D. (2020). Macroeconomic effects and frailties in the resolution of non-performing loans. J. Bank. Financ..

[26] Arping S. (2014). Credit protection and lending relationships. J. Financ. Stab..

[27] Buston C.S. (2016). Active risk management and banking stability. J. Bank. Financ..

[28] Beyhaghi M., Massoud N., Saunders A. (2017). Why and how do banks lay off credit risk? The choice between retention, loan sales and credit default swaps. J. Corp. Financ..

[29] Gersbach H., Rochet J.C. (2017). Capital regulation and credit fluctuations. J. Monet. Econ..

[30] Gefang D., Koop G., Potter S.M. (2011). Understanding liquidity and credit risks in the financial crisis. J. Empir. Financ..

[31] De Socio A. (2013). The interbank market after the financial turmoil: Squeezing liquidity in a “lemons market” or asking liquidity “on tap”. J. Bank. Financ..

[32] Beirne J. (2012). The EONIA spread before and during the crisis of 2007-2009: The role of liquidity and credit risk. J. Int. Money Financ..

[33] Ippolito F., Peydró J.L., Polo A., Sette E. (2016). Double bank runs and liquidity risk management. J. Financ. Econ..

[34] Imbierowicz B., Rauch C. (2014). The relationship between liquidity risk and credit risk in banks. J. Bank. Financ..

[35] Chen H.J., Lin K.T. (2016). How do banks make the trade-offs among risks? The role of corporate governance. J. Bank. Financ..

[36] Bouslimi J., Hakimi A., Zaghdoudi T., Tissaoui K. (2024). The complex relationship between credit and liquidity risks: A linear and non-linear analysis for the banking sector. Hum. Soc. Sci. Commun..

[37] Aydemir R., Guloglu B. (2017). How do banks determine their spreads under credit and liquidity risks during business cycles?. J. Int. Financ. Mark. Inst. Money.

[38] Djebali N., Zaghdoudi K. (2020). Threshold effects of liquidity risk and credit risk on bank stability in the MENA region. J. Policy Model..

[39] Abdesslem R.B., Chkir I., Dabbou H. (2022). Is managerial ability a moderator? The effect of credit risk and liquidity risk on the likelihood of bank default. Int. Rev. Financ. Anal..

[40] Zhang M.H., He J.M., Li S.W. (2018). Interbank lending, network structure and default risk contagion. Physical A.

[41] Chen T.Q., Wang Y.T., Zeng Q.R., Luo J. (2020). Network model of credit risk contagion in the interbank market by considering bank runs and the fire sale of external assets. Physical A.

[42] Denbee E., Julliard C., Li Y., Yuan K. (2021). Network risk and key players: A structural analysis of interbank liquidity. J. Financ. Econ..

[43] Chen Y. (2022). Bank interconnectedness and financial stability: The role of bank capital. J. Financ. Stab..

[44] Bookstaber R., Paddrik M., Tivnan B. (2018). An agent-based model for financial vulnerability. J. Econ. Interact. Coord..

[45] Paltalidis N., Gounopoulos D., Kizys R., Koutelidakis Y. (2015). Transmission channels of systemic risk and contagion in the European financial network. J. Bank. Financ..

[46] Silva T.C., da Silva Alexandre M., Tabak B.M. (2018). Bank lending and systemic risk: A financial-real sector network approach with feedback. J. Financ. Stab..

[47] Li S., Wang H., Liu X. (2022). Research on the effect of systemic risk prevention and control policy based on dynamic multilayer network of banks. J. Ind. Eng. Manag..

[48] Hu L.Q., Gan Y.R., Wen H.L. (2023). Do we need to consider multiple inter-bank linkages for systemic risk in China’s banking industry? Analysis based on the multilayer network. Financ. Res. Lett..

[49] Gao Q., Fan H. (2025). Research on systemic risk of multi-layered financial networks based on risk-averse behavior of agents. J. Ind. Eng. Manag..

[50] Tedeschi G., Vidal-Tomás D., Delli-Gatti D., Gallegati M. (2021). The macroeconomic effects of default and debt restructuring: An agent based exploration. Int. Rev. Econ. Financ..

[51] Gurgone A., Iori G. (2022). Macroprudential capital buffers in heterogeneous banking networks: Insights from an ABM with liquidity crises. Eur. J. Financ..

[52] Adao L.F.S., Silveira D., Ely R.A., Cajueiro D.O. (2022). The impacts of interest rates on banks’ loan portfolio risk-taking. J. Econ. Dyn. Control.

[53] Grilli R., Tedeschi G., Gallegati M. (2014). Bank interlinkages and macroeconomic stability. Int. Rev. Econ. Financ..

[54] Gatti D.D., Gallegati M., Greenwald B., Russo A., Stiglitz J.E. (2010). The financial accelerator in an evolving credit network. J. Econ. Dyn. Control.

